# Role of Local Excision for Suspected Regrowth in a Watch and Wait Strategy for Rectal Cancer

**DOI:** 10.3390/cancers14133071

**Published:** 2022-06-23

**Authors:** Barbara M. Geubels, Vincent M. Meyer, Henderik L. van Westreenen, Geerard L. Beets, Brechtje A. Grotenhuis, on behalf of the Dutch Watch and Wait Consortium

**Affiliations:** 1 Department of Surgery, Netherlands Cancer Institute—Antoni van Leeuwenhoek, Postbox 90203, 1006 BE Amsterdam, The Netherlands; b.geubels@nki.nl; 2 Department of Surgery, Catharina Hospital, Postbox 1350, 5602 ZA Eindhoven, The Netherlands; g.beets@nki.nl; 3 GROW School for Oncology and Developmental Biology, Maastricht University, 6200 MD Maastricht, The Netherlands; vincentmeyer@gmail.com; 4 Department of Surgery, University Medical Centre Groningen, Postbox 30.001, 9700 RB Groningen, The Netherlands; 5 Department of Surgery, Isala Hospitals, Postbox 10400, 8000 GK Zwolle, The Netherlands; h.l.van.westreenen@isala.nl

**Keywords:** rectal cancer, Watch and Wait, local regrowth, local excision, oncological outcome, organ preservation

## Abstract

**Simple Summary:**

Rectal cancer patients with a clinical complete response to neoadjuvant treatment are eligible for Watch and Wait as an alternative to total mesorectal excision. However, in patients with local regrowth, major surgery is still the standard of care. The present study evaluates the role of local excision for suspected local regrowth in a large Watch and Wait cohort, in terms of long-term outcomes. This study shows excellent overall survival and a good organ preservation rate. Patients who developed locoregional recurrence after initial local excision for regrowth were all successfully treated with salvage surgery. This study shows that local excision can provide maintenance of organ preservation without an obvious compromise in surgical or oncological safety. Local excision for suspected regrowth in patients following Watch and Wait can be a safe alternative for total mesorectal excision in selected patients with a strong wish to preserve their rectum.

**Abstract:**

Rectal cancer patients with a clinical complete response to neoadjuvant (chemo)radiation are eligible for Watch and Wait (W&W). For local regrowth, total mesorectal excision (TME) is considered the standard of care. This study evaluated local excision (LE) for suspected local regrowth. From 591 patients prospectively entered into a national W&W registry, 77 patients with LE for regrowth were included. Outcomes analyzed included histopathologic findings, locoregional recurrence, long-term organ preservation, and colostomy-free and overall survival. In total, 27/77 patients underwent early LE (<6 months after neoadjuvant radiotherapy) and 50/77 underwent late LE (≥6 months). Median follow-up was 53 (39–69) months. In 28/77 patients the LE specimen was histopathologically classified as ypT0 (including 9 adenomas); 11/77 were ypT1, and 38/77 were ypT2–3. After LE, 13/77 patients with ypT2–3 and/or irradical resection underwent completion TME. Subsequently, 14/64 patients without completion TME developed locoregional recurrence, and were successfully treated with salvage TME. Another 8/77 patients developed distant metastases. At 5 years, overall organ preservation was 63%, colostomy-free survival was 68%, and overall survival was 96%. There were no differences in outcomes between early or late LE. In W&W for rectal cancer, LE can be considered as an alternative to TME for suspected regrowth in selected patients who wish to preserve their rectum or avoid colostomy in distal rectal cancer.

## 1. Introduction

In recent years, non-operative treatment strategies in excellent responders to neoadjuvant therapy have gained popularity because of the improved functional outcomes and quality of life. Rectal cancer patients with a clinical complete response at restaging after neoadjuvant therapy can be safely monitored in a “Watch and Wait” (W&W) protocol [1,2]. Most series report a local regrowth rate of 20–30% but, reassuringly, these patients are amenable to a delayed total mesorectal excision (TME) without any obvious compromise in oncological outcomes and surgical safety [3,4,5,6]. With the goal in mind of obtaining better functional results and continuing an organ-preserving approach, the question is whether small luminal regrowths can also be treated with a transanal local excision (LE).

LE has been part of organ preservation in certain strategies where in relatively early rectal tumors the scar or small remnant after chemoradiation has been removed as a planned procedure [4,7,8,9,10]. With this strategy, preservation of the rectum can be ensured in a high number of cases without compromising locoregional control [11,12]. LE can also be performed more selectively for tumors that, while responding well, do not show the typical picture of a clinical complete response (also labeled as near-complete response). Yet another approach in organ preservation is to perform LE when a regrowth occurs, or is suspected, during follow-up in the W&W strategy [10]. Tumor regrowth is reappearance of neoplasia at the site of the primary tumor after a clinical complete response to neoadjuvant therapy in a W&W program, where TME has been omitted—in contrast to the definition of locally recurrent rectal cancer, which occurs after TME, and is much more difficult to treat, with a more reserved prognosis. In the use of LE for regrowth in a W&W approach, there is nevertheless some concern that there may be more extended residual tumor through the bowel wall and in the mesorectal lymph nodes—especially in patients who originally had a more advanced tumor. LE would then expose patients to undertreatment, with risks of recurrence [13,14,15,16].

To date, two studies have reported on the oncological outcomes of LE for regrowth [6,17]. The aim of the present study was to evaluate the outcomes of patients who underwent LE for suspected regrowth in a large W&W cohort in the Netherlands. 

## 2. Materials and Methods

### 2.1. Patient Data 

Patients with rectal cancer who followed a W&W strategy after neoadjuvant (chemo)radiation between 2004 and 2017 were prospectively included in a local study from the Maastricht University Medical Center, approved by the local institutional review board and registered on ClinicalTrials.gov since 2009 (NCT00939666 and NTC02278653). W&W patients from 2017 onwards were prospectively included in the national multicenter registry study “Wait-and-see” Policy for Complete Responders After Chemoradiotherapy for Rectal Cancer (ClinicalTrials.gov NCT03426397), coordinated by the Netherlands Cancer Institute in Amsterdam. For quality assurance purposes, the response evaluation data from the multicenter study from 2017 onwards were reassessed by the coordinating center, all participating centers were site-visited, and teaching sessions were organized. Written informed consent was obtained from all patients.

### 2.2. Neoadjuvant Treatment, Selection, and Follow-Up in the W&W Program

Patients were treated with long-course chemoradiotherapy (28 fractions of 1.8 Gy or 25 fractions of 2.0 Gy) with twice-daily bolus capecitabine 825 mg/m^2^, or with short-course radiotherapy (5 fractions of 5 Gy) followed by a prolonged waiting interval. Restaging was performed with digital rectal examination (DRE), endoscopy, and MRI 8–12 weeks after the radiotherapy. Patients with a clinical complete response entered the W&W protocol. Patients with a very good response and no signs of residual tumor but not the typical image of a complete response—such as a small superficial ulceration—could also enter the W&W protocol under the label near-complete response. Patients with persistent near-complete response at repeated assessment were recommended for TME, but were also given the option of LE if technically feasible. 

Standard follow-up consisted of computed tomography scan, colonoscopy, and carcinoembryonic antigen measurements according to national guidelines, for 5 years. The additional follow-up in the W&W program consisted of DRE, endoscopy, and MRI including diffusion-weighted imaging every 3 months for the first 2 years, and 6-monthly thereafter for up to 5 years.

### 2.3. Diagnosis and Treatment of Regrowth

Regrowth during follow-up in the W&W program was preferably proven by biopsy, but sometimes there were only suspicious findings on endoscopy or MRI. Patients were offered TME as the standard treatment option, and LE by performing transanal endoscopic microsurgery (TEM) or transanal minimally invasive surgery (TAMIS) if the local regrowth was small and without signs of involved lymph nodes on MRI (or as a diagnostic procedure when the regrowth was not proven). Ultimately, the choice between TME or LE depended on the patient’s preference, comorbidities, and the advice of the multidisciplinary team. When patients underwent LE for regrowth, and the histological examination of the resection specimen showed ypT0 or ypT1 with free resection margins (>1 mm), the patients continued follow-up in the W&W protocol. Completion TME was advised when the resection specimen showed ypT2–3 or an irradical resection (i.e., microscopic margin involvement with the tumor either laterally or at a deep resection surface (R1), or a too-fragmented resection specimen).

### 2.4. Outcomes

Reported outcomes were histopathologic findings after LE, locoregional-recurrence-free survival after LE, overall survival, organ preservation rate, colostomy-free survival and 90-day morbidity after LE, completion TME, and salvage TME. Locoregional recurrence after LE was specified as any luminal or (nodal) mesorectal recurrent disease within the pelvis. Subgroup analyses were performed for “early” and “late” LE, with a cutoff at 6 months after the last radiation, to capture any differences between persistent disease (i.e., near-complete response that never evolved into a clear clinical complete response) and regrowths after an initial clinical complete response. 

### 2.5. Data Analysis

Statistical analysis was performed with IBM SPSS statistics version 27. Descriptive statistics were provided for baseline and treatment variables, as well as outcome measures. Follow-up time was calculated from primary MRI until the date of the last follow-up moment for all outcome measures, except for the locoregional-recurrence-free interval, which was calculated from LE until locoregional recurrence or death. Kaplan–Meier survival methods were used for survival analysis.

## 3. Results

### 3.1. Patient Characteristics

**Figure 1:**
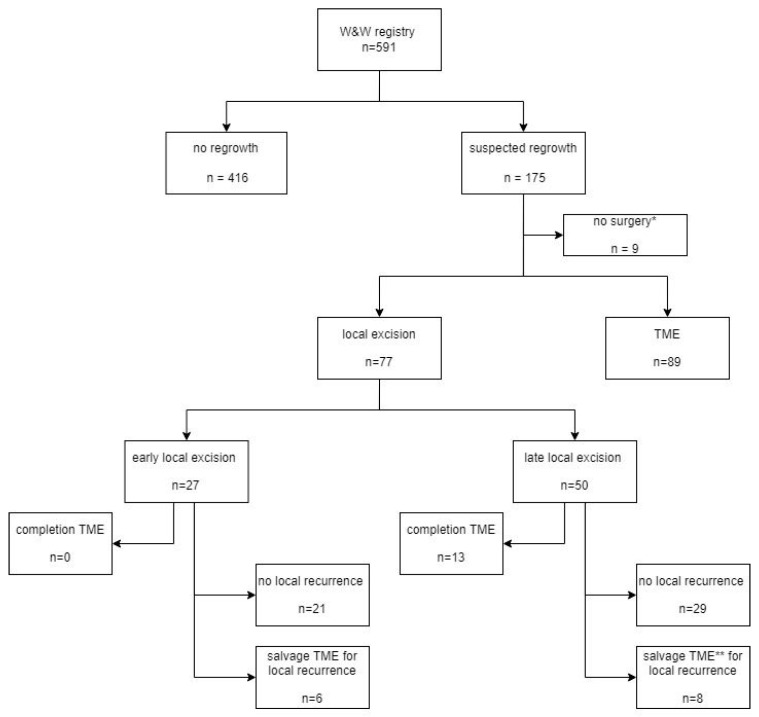
Flowchart depicting patient flow. Abbreviations: W&W = Watch and Wait; TME = total mesorectal excision; * due to widespread metastases, frailty or patient preferences; ** *n* = 1 salvage TME after second local excision.

Between 2004 and 2019, 591 rectal cancer patients were prospectively registered in the W&W registry, with a minimum follow-up of two years (Figure 1); 68% of the patients were male, and their median age was 65 years. Most patients were diagnosed with cT3–4 rectal cancer (81%) and cN+ disease (72%). The majority of the patients (92%) were treated with neoadjuvant chemoradiation. The rectal tumor was located in the distal rectum (i.e., <5 cm from the anal verge) in 65% of patients.

During follow-up, 166 patients underwent surgical treatment for a suspected regrowth, of whom 77 patients underwent LE and were included in the present study (Figure 1). Baseline characteristics are shown in Table 1. The median time (IQR) between the end of neoadjuvant treatment and first restaging was 8 (7–11) weeks. A clinical complete response was seen at that time in 26 patients (34%), while 51 patients (66%) were considered as having a clinical near-complete response. 

The median time (IQR) between the end of (chemo)radiation and LE was 7 (5–11) months. LE was performed within 6 months in 27/77 (35%) patients, and after 6 months in 50/77 (65%) patients. There were 23/27 (85%) patients with a near-complete response at first restaging in the early LE group, in contrast to 28/50 (56%) patients in the late LE group.

### 3.2. Histology after LE

Histological results are shown in Table 2. Overall, in 28/77 (36%) patients the LE specimen was histologically classified as ypT0, with adenoma in 9, fibrosis or inflammation in 8, and nonspecific findings in 11. In 20 of these 28 ypT0 cases, an endoscopic biopsy had been performed preoperatively, of which 8 were suspected for malignancy. 

Overall, the LE specimens revealed ypT1 tumors in 11 (14%) patients, ypT2 in 32 (42%) patients, and ypT3 in 6 (8%) patients. In 37 of the 49 patients with ypT1–3, an endoscopic biopsy had been performed prior to the LE, of which 32 were suspected for malignancy.

The margins were clear of tumor cells in 73/77 (95%) patients. One patient had tumor involvement at the deep margin, and another at the deep and lateral margin. In two patients, the resection specimen was too fragmented and not possible to reconstruct. Therefore, we concluded that in four patients a radical resection of the lesion was not achieved. 

In the early LE group there were relatively more ypT0 cases than in the late group: 15/27 (56%) versus 13/50 (26%). In the early LE group, no completion TME was performed, including the 8 patients with ypT2 or ypT3 tumors who declined completion surgery. In the late LE group, 13 patients (1 patient with a ypT1 tumor and irradical resection, 8 patients with ypT2 tumors, and 4 patients with ypT3 tumors) underwent completion TME.

### 3.3. Long-Term Outcomes 

The median follow-up time (IQR) was 53 (39–69) months. Three patients died during follow-up: one patient from unrelated disease and two patients from metastatic disease. The 2-year and 5-year overall survival was 99% and 96%, respectively. Overall, 2-year and 5-year locoregional-recurrence-free survival after LE was 85% and 71%, respectively. Fifteen out of all seventy-seven patients (19%) developed a locoregional recurrence. Of 49/77 patients with actual regrowth (ypT1–3), 11 (22%) patients experienced locoregional recurrence, with 2-year and 5-year locoregional-recurrence-free survival after LE of 74% and 62%, respectively.

As shown in Table 3, 14 out of 64 (22%) patients who did not undergo completion TME developed locoregional recurrence: 10 luminal regrowths, 3 nodal regrowths, and 1 nodal regrowth combined with combined liver metastases. All of these 14 patients underwent successful salvage TME with a radical resection. Of the 13 patients who underwent completion TME, 1 patient developed an iliac lymph node recurrence and 2 patients developed distant metastases. After TME surgery, the median follow-up time (IQR) was 28 months (19–42). 

**Figure 2:**
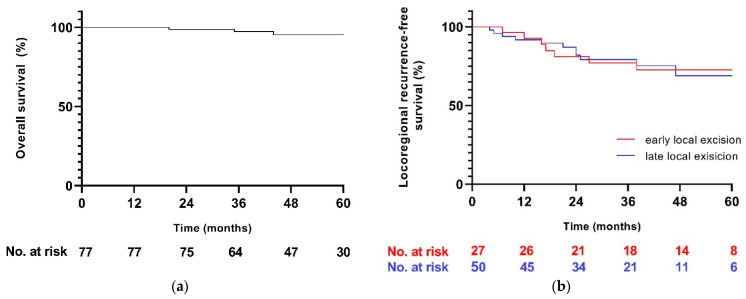
Kaplan–Meier curves for (**a**) overall survival and (**b**) locoregional-recurrence-free survival for the early and late local excision groups.

The remaining 50 patients who did not undergo completion or salvage TME continued follow-up in the W&W protocol, and 5 patients developed distant metastases at a later stage. There were no significant differences in locoregional recurrence between the early and late LE groups (Figure 2). 

The overall organ preservation rate at 2 and 5 years was 79% and 63%, respectively. Colostomy-free survival was 81% at 2 years and 68% at 5 years.

### 3.4. Complications after LE and TME Surgery

After LE, 6/77 (8%) patients had Clavien–Dindo grade 2 or higher complications. Of these, one patient was admitted to the intensive care unit secondary to heart failure, and five had surgical complications: two postoperative bleedings, two wound dehiscences, and one patient with an abscess. No interventions were required. 

After completion TME, 3/13 (23%) patients experienced a Clavien–Dindo grade 2 or higher complication: one pneumonia, one prolonged ileus, and one anastomotic leakage requiring surgery. 

After salvage TME, 3/14 (21%) patients experienced a complication classified as Clavien–Dindo > 2. One patient developed pneumonia treated with intravenous antibiotics, an abscess was treated with antibiotics and, finally, one patient developed an anastomotic leak, for which a reoperation was performed.

## 4. Discussion

The aim of the present study was to report the outcomes of LE for proven or suspected regrowths in a W&W approach, with the goal of maintaining organ preservation. The overall 5-year survival of 96% in this selected patient group was good. A locoregional recurrence occurred in 22% of the large group of patients who did not undergo completion TME, and in 8% of the small group of patients who underwent completion TME. All local recurrences after LE were salvaged with TME surgery without further recurrence. With a 68% colostomy-free survival and 63% organ preservation rate at 5 years, LE can provide maintained organ preservation in a substantial number of patients with a proven or suspected regrowth, without any obvious compromise in surgical or oncological safety in this selected group of patients following W&W.

Many centers favor TME resection as locoregional treatment for the 30% of patients with a regrowth, because LE may be considered oncologically suboptimal for a tumor that was T3 or N+ on baseline imaging. There are two reports on the use of LE for regrowths in a W&W protocol. In the series reported by Fernandez et al., 32% of regrowths were treated with LE, with a local recurrence rate of 14%. In the series reported by Van der Sande et al., 30% of regrowths were treated with LE, with a local recurrence rate of 8% [6,17]. The present study showed a higher local recurrence rate of 19%, most likely related to the more advanced stage at baseline staging and our more liberal use of LE, as 44% of patients with regrowth underwent LE (Figure 1). Ultimately, the most important oncological question remains whether a local recurrence after LE will lead to uncontrolled locoregional disease. Both other studies reported that all local recurrences were salvaged with TME. In the present study, all local recurrences after LE underwent radical resection after TME surgery, with no further local recurrence in the follow-up. Finally, the overall long-term outcomes of the present cohort were very good, and compare favorably to series that report on the outcomes of immediate TME surgery as treatment for regrowths in a W&W approach [5,16]. 

The present study also included LE for lesions that were suspicious, but not proven to be malignant. In some patients with a near-complete response, remnant lesions may not disappear with further follow-up. A negative biopsy does not rule out residual tumor, creating a diagnostic dilemma [18]. In other patients, adenomatous lesions can appear in the scar during follow-up, with a biopsy showing low- or high-grade dysplasia, but no invasive cancer. Again, this creates the same diagnostic dilemma: should we wait longer, or should we remove the lesion? A diagnostic LE can provide a definitive answer, while also providing the chance of being therapeutic in case of a small residual tumor. In the present series, diagnostic LE in terms of ypT0 histological outcomes was much more common in the first 6 months of follow-up.

Some surgeons favor routine LE of the scar after chemoradiation, with the goal of removing any potential small tumor remnants and avoiding regrowths. Studies on this strategy generally report low local recurrence rates, such as the reports by d’Alimonte et al. and Bushati et al., who both reported an 8% local recurrence rate [11,12]. The downside of conducting routine LE is that some reports have shown a higher complication rate compared to LE without preceding radiotherapy, although in the present study only 8% of patients had Clavien–Dindo > 2 complications. Additionally, worse functional outcomes of LE after radiotherapy have been reported [19,20,21,22,23]. As the majority of patients with a clinical complete or near-complete response have no residual tumor, they will gain no benefit from routine LE, while increasing their chances of anorectal dysfunction. In a recent review on the role of LE for organ preservation after radiotherapy, Perez et al. noted that most surgeons have moved away from routine LE, and made a case for much more selective use [24].

This study has limitations. Some of the concepts and practices of W&W evolved during the 15-year study period. Initially, only patients with a typical clinical complete response were included, and the concept of near-complete response developing into a complete response with further observation gradually led to more inclusion of near-complete responders. Likewise, the use of LE for suspected or proven regrowths gradually increased over time, as a result of a favorable experience in highly selected patients in the early study period [6]. Therefore, only a small group of patients with LE for suspected regrowth completed the 5-year follow-up.

In addition, this is a database-based registry study with variability between participating centers. The indication for LE was at the discretion of the local colorectal team, and was not well documented. It was not possible to reconstruct the exact considerations in the 44% of patients in whom it was decided to perform LE rather than TME. In general, the lesion had to be small, with no evidence of mesorectal lymph nodes or deposits, preferably not located anteriorly (i.e., close to the prostate or vaginal wall) and, provided LE was technically feasible, the patient had to express a strong wish for organ preservation.

Finally, in a W&W strategy, LE can be an alternative to TME surgery for a suspected or proven regrowth in patients with a strong wish to preserve their rectum or avoid a colostomy in distal rectal cancer. However, an important issue regarding the applicability of the results of this study in daily clinical practice is that patients in the present cohort were generally treated by dedicated colorectal teams and in highly experienced centers for W&W. 

Although in the present series all local recurrences after LE could successfully be salvaged with TME without further local recurrence, caution is required given the limitations of the current study. Shared decision making, balancing the functional benefit of LE and organ preservation against the (small) potential oncological risk, is essential.

## 5. Conclusions

In conclusion, LE for suspected regrowth can provide maintained organ preservation in a substantial number of patients following W&W, without any obvious compromise in surgical or oncological safety. In a W&W program for rectal cancer, LE can be considered as an alternative to TME for suspected regrowth in selected patients who wish to preserve their rectum or avoid a colostomy in distal rectal cancer. 

## Figures and Tables

**Table 1:** Baseline characteristics of *n* = 77 patients who underwent local excision for suspected regrowth within the W&W cohort.

	*n* = 77
Age, median (range), years	66 (43–87)
Sex, *n* (%)	
Male	57 (74)
Female	20 (26)
Clinical T stage, *n* (%)	
T1	6 (8)
T2	19 (25)
T3	47 (61)
T4	5 (7)
Clinical N stage, *n* (%)	
N0	33 (43)
N1	25 (33)
N2	19 (25)
Distance anal verge, mean (SD), cm	2.8 (2.8)
<5 cm, *n* (%)	59 (77)
≥5 cm, *n* (%)	18 (23)
Neoadjuvant therapy, *n* (%)	
- Chemoradiotherapy	66 (86)
- Short-course radiotherapy + interval	11 (14)

**Table 2:** Histological results after early (<6 months after last radiation) and late (≥6 months) local excision.

	Early LE	Late LE
	*n* = 27	*n* = 50
ypT stage, *n* (%)		
ypT0	15 (56)	13 (26)
ypT1	4 (15)	7 (14)
ypT2	7 (26)	25 (50)
ypT3	1 (4)	5 (10)
Radical resection, *n* (%)	27 (100)	46 (92)

Abbreviations: LE = local excision.

**Table 3:** Oncological outcomes and organ preservation subdivided for patients with and without completion TME after local excision.

	No Completion TME	Completion TME
	*n* = 64	*n* = 13 *
Local recurrence only, *n* (%)	13 (20%)	*na*
Luminal, *n*	10	
Nodal, *n*	3	
Local + systemic recurrence, *n* (%)	1 (2%)	*na*
Systemic recurrence only, *n* (%)	5 (8%)	2 (15%)
Salvage TME, *n* (%)	14 (22%)	*na*
Local recurrence after TME, *n* (%)	0	1 (8%)
Colostomy rate, *n* (%)	10 (13%)	11 (85%)
Alive	62 (97%)	12 (92%)

Abbreviations: TME = total mesorectal excision; * *n* = 1 patient with ypT1 and irradical resection, *n* = 12 patients with ypT2–3; *na* = not available.

## Data Availability

Study data are available for review, upon reasonable request only.
